# Supplementary material for: Mechanochemical Treatment in High-Shear Thermokinetic Mixer as an Alternative for Tire Recycling
Source: Polymers (Basel). 2022 Oct 19;14(20):4419. doi: 10.3390/polym14204419 (PMC9610282; doi:10.3390/polym14204419)
Supplement: Supplementary file 1 [file polymers-14-04419-s001.zip › polymers-1981177-supplementary.pdf]

Supplementary files for:

Mechanochemical Treatment in High-Shear Thermokinetic Mixer as an Alternative for Tire Recycling

Otávio Bianchi *, Patrícia Bereta Pereira and Carlos Arthurs Ferreira

LAPOL/PPGE3M/Universidade Federal do Rio Grande do Sul, Av. Bento Gonçalves 9500, Porto Alegre 90010-150, Brazil
* Correspondences: otavio.bianchi@gmil.com

This supplementary file shows the results of the EDX analysis, which was used to identify the main elements present in the GTR sample qualitatively.

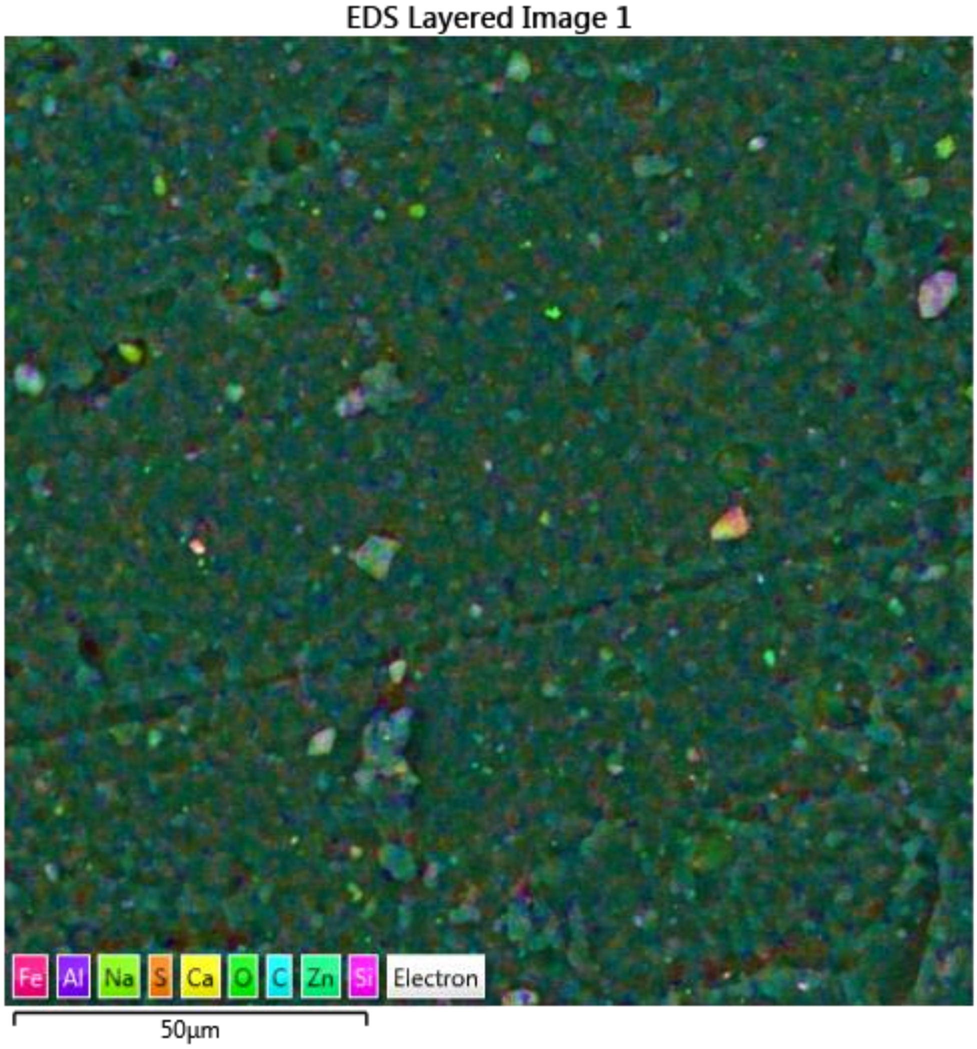

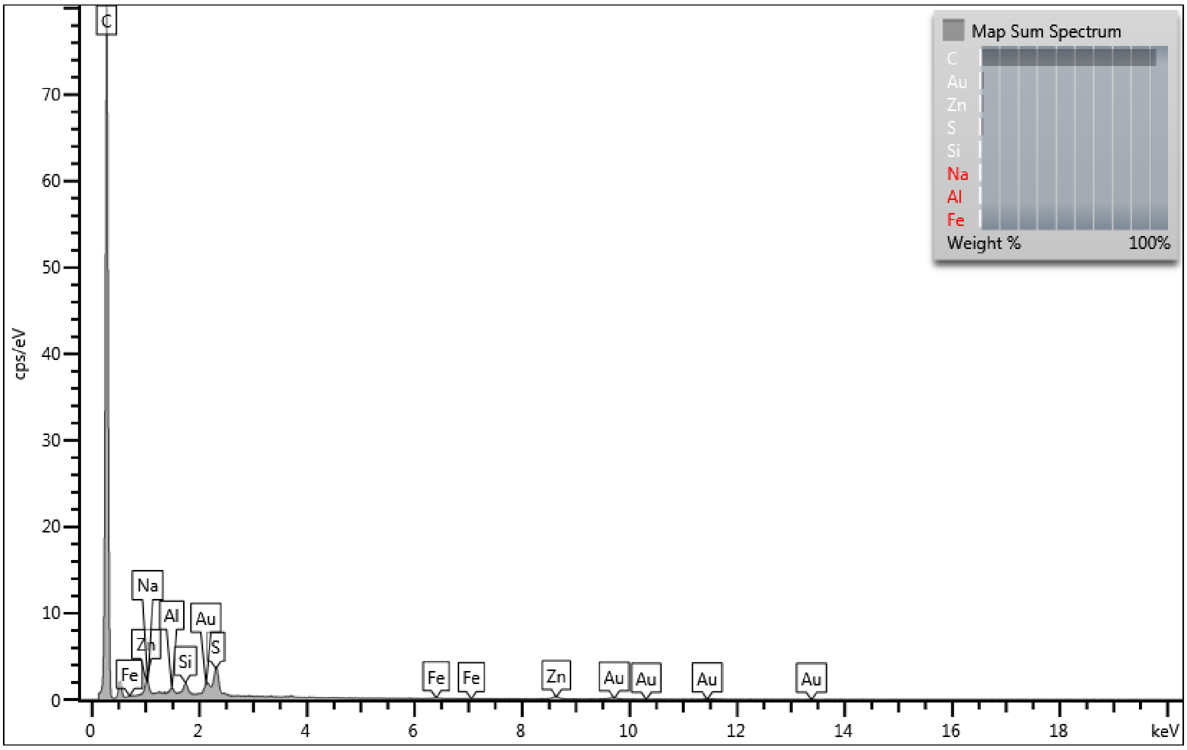

Figure S1. EDS analysis for GTR sample.

Electron Image 1

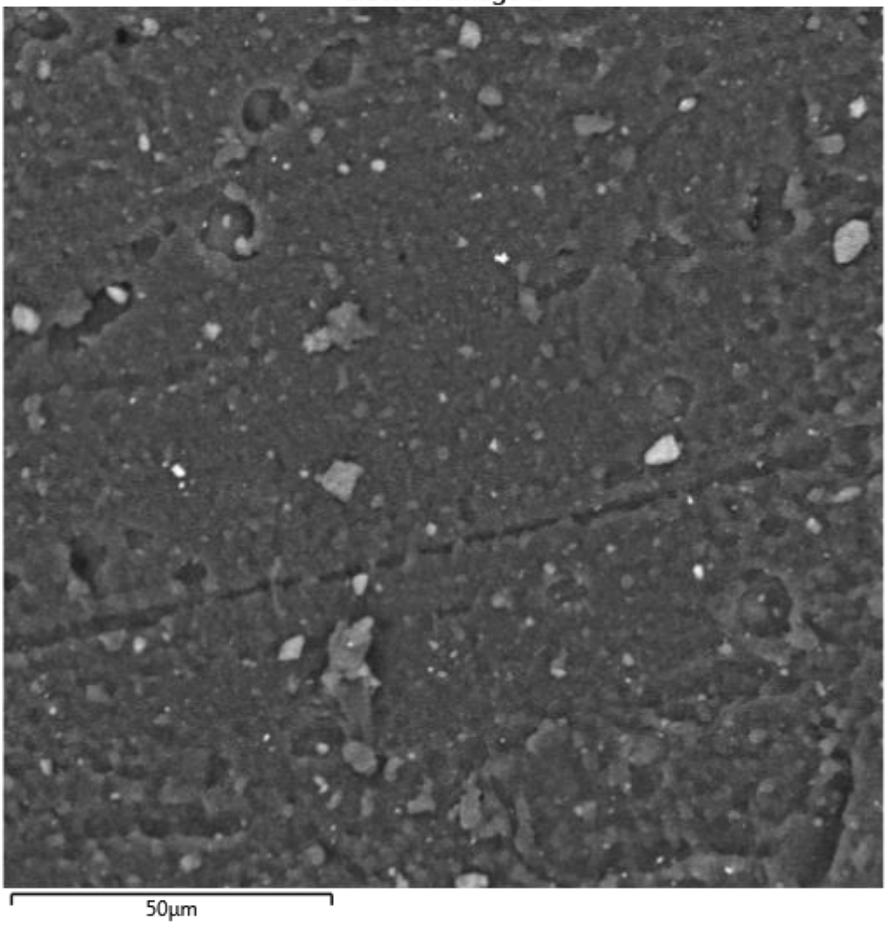

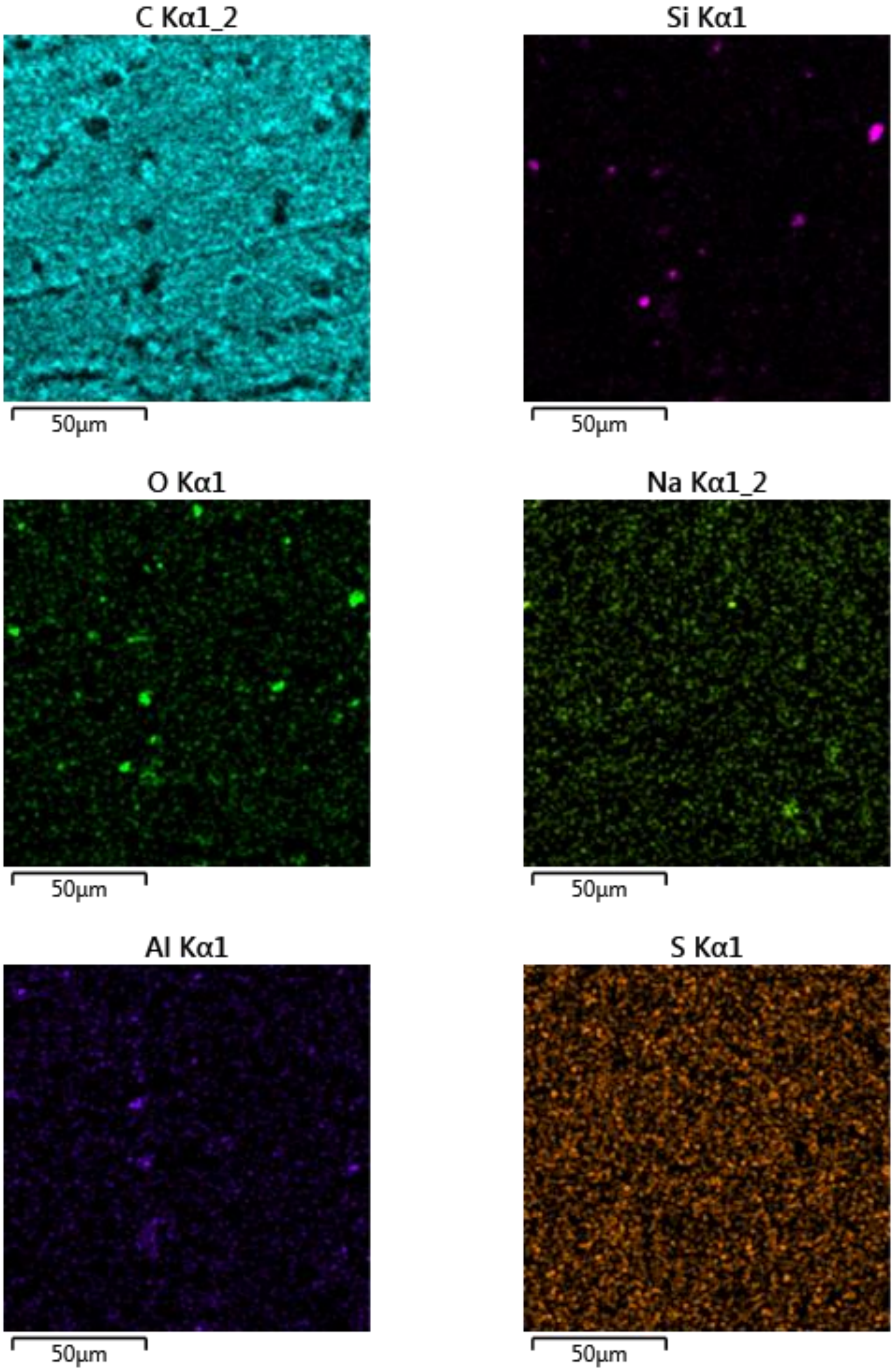
C Kα1_2
50μm
Si Kα1
50μm
O Kα1
50μm
Na Kα1_2
50μm
Al Kα1
50μm
S Kα1
50μm

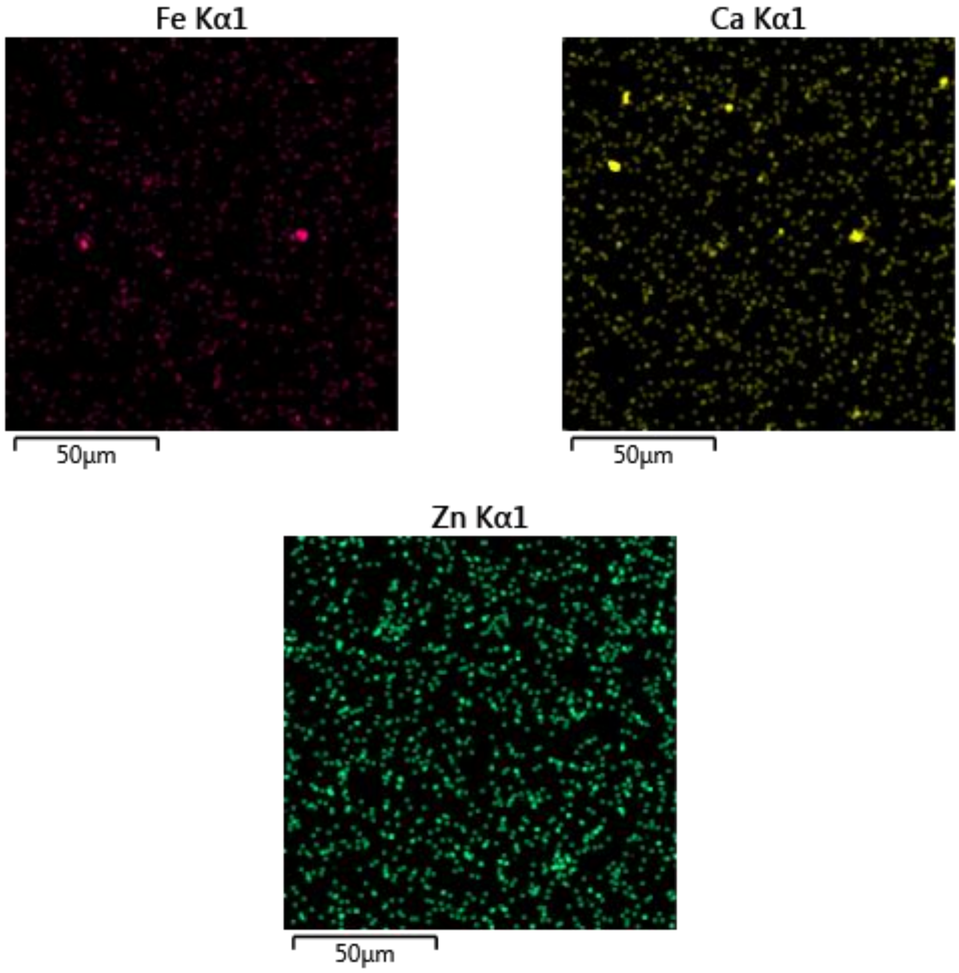

Figure S2. Compositional map for GTR sample.
